# Understanding the mediating role of pharmaceutical policymaking attitudes between personal attributes and public service motivation

**DOI:** 10.1016/j.rcsop.2026.100722

**Published:** 2026-02-18

**Authors:** Chadia Haddad, Hala Sacre, Jihan Safwan, Deema Rahme, Aline Hajj, Jenny Elia, Joya El Ghawi, Lina Haidar, Lama Dimachkieh, Mahmoud Nasrallah, Sukaina Basma, Pascale Salameh

**Affiliations:** a Institut National de Santé Publique, d'Épidémiologie Clinique et de Toxicologie-Liban (INSPECT-LB), Beirut, Lebanon; b Research Department, Psychiatric Hospital of the Cross, Jal Eddib, Lebanon; c Faculty of Public Health, Lebanese University, Fanar, Lebanon; d Inserm U1094, IRD UMR270, EpiMaCT Epidemiology of Chronic Diseases in Tropical Zone, University of Limoges, Limoges, France; e School of Pharmacy, Lebanese International University, Beirut, Lebanon; f Pharmacy Practice Department, Faculty of Pharmacy, Beirut Arab University, Beirut, Lebanon; g Faculté de Pharmacie, Université Laval, Québec, Canada.; h Oncology Division, CHU de Québec Université Laval Research Center, Québec, Canada; i Lebanese Pharmacy Students' Association, Beirut, Lebanon; j Faculty of Pharmacy, Lebanese University, Hadat, Lebanon; k Gilbert and Rose-Marie Chagoury School of Medicine, Lebanese American University, Beirut, Lebanon; l Department of Primary Care and Population Health, University of Nicosia Medical School, 2417 Nicosia, Cyprus

**Keywords:** Public service motivation, Policymaking perception, Pharmacy practice, Leadership, Strategic thinking, Self-efficacy, Mediation analysis, Pharmacy education

## Abstract

**Background:**

The mediating role of perceptions of pharmaceutical policymaking on the relationship between personal attributes and public service motivation (PSM) remains unexplored, particularly among early-career pharmacists in developing countries such as Lebanon. This study aimed to explore the relationship between personal and professional attributes, specifically leadership, strategic thinking, and self-efficacy, and PSM. It also assessed whether perceptions of pharmaceutical policymaking mediate these relationships and whether these pathways vary across different pharmacist personal attribute profiles.

**Methods:**

This cross-sectional study was conducted from December 2023 to May 2024 and included early-career pharmacists. This study used validated scales to assess personal and professional attributes.

**Results:**

Among 504 pharmacists, 32% were early career pharmacists, 30% were senior students, and the rest were fourth-year or earlier pharmacy students. Participants were grouped into three profiles based on personal attributes: strong, moderate, and weak. Authentic leadership was positively correlated with generalized self-efficacy (*r* = 0.465, *p* < 0.001), research self-efficacy (*r* = 0.273, *p* < 0.001), strategic thinking (*r* = 0.489, *p* < 0.001), and public service motivation (*r* = 0.569, p < 0.001). Regression analysis showed that leadership (β = 0.006, *p* < 0.001), generalized self-efficacy (β = 0.007, p < 0.001), research self-efficacy (β = 0.001, *p* = 0.004), and strategic thinking (β = 0.007, p < 0.001) positively predicted PSM. Mediation analysis indicated significant direct effects of these traits on PSM in the total sample (all *p* < 0.05). When the cluster group was considered as the independent variable, Pharmaceutical Policymaking Perception Scale partially mediated the relationship with PSM, with significant direct (β = −5.071, *p* = 0.009) and indirect effects (β = −1.209, *p* = 0.006).

**Conclusion:**

This research found that leadership, strategic thinking, and generalized and research self-efficacy were positively associated with public service motivation, while pharmaceutical policymaking was inversely related to it. Perception of pharmaceutical policymaking partially mediated the effect of weaker cluster membership on public service motivation. In view of the paucity of research in this area, conducting similar studies in diverse settings would help confirm the current results and further consolidate the causal relationships between these concepts. Meanwhile, education and research should prioritize early-career pharmacists' personal attributes to empower them in optimizing their profession and enhancing patient care.

## 1 Background

Public service motivation (PSM) has become crucial to understanding the behavior and performance of public sector employees, including healthcare professionals such as pharmacists. Within pharmacy leadership, PSM reflects the altruistic desire of pharmacists to serve the public interest and contribute to the well-being of patients, regardless of financial incentives.^1^ Factors such as attraction to policymaking, desire for strategic impact, compassion, and self-sacrifice have all been linked to PSM.^2^ In pharmacy practice, altruism and PSM are related but distinct concepts. Whether these concepts are overlapping or distinct is not always evident, as the terms are often used interchangeably. Altruism is generally defined as a selfless concern for others that can be a component of PSM. It represents a specific type of motivation centered on serving the public interest. PSM encompasses a broader set of motives, which may not always be entirely altruistic, such as a commitment to public principles and values and a desire to improve society.^3^

Previous research suggests that both individual characteristics (e.g., personality traits and psychological needs)^4^ and organizational factors (e.g., work environment)^5^ significantly influence the motivation of healthcare professionals, including pharmacists, as caregivers and service providers.^6^, ^7^, ^8^ Recent studies have highlighted the complex interplay between personal attributes, organizational perceptions, and PSM in various contexts.^9^ Leadership and strategic thinking have been particularly identified as essential contributors to PSM, while self-efficacy, defined as an individual's belief in their ability to successfully perform tasks and achieve desired outcomes, has shown varying associations with motivation in different settings.^2^, ^9^, ^10^ However, differences in the perceived importance of these attributes between early-career pharmacists and more experienced pharmacists^11^ underscore the need to consider psychological constructs as part of the PSM concept among healthcare professionals.^8^

Despite these insights, the role of pharmaceutical policymaking perception as a mediator between personal attributes and PSM remains unexplored, particularly among early-career pharmacists in developing countries like Lebanon. The pharmacy profession in Lebanon has been severely affected by multiple crises, starting with political instability, the COVID-19 pandemic, and economic deterioration. An oversupply of non-specialized pharmacists, a lack of specialized pharmacists, and inadequate policy development and implementation have further strained practicing pharmacists and the pharmacy sector in general.^12^, ^13^ Pharmacists' perceptions of policymaking engagement represent an important cognitive and professional mechanism that may connect individual attributes with motivational outcomes: a recent study by our team found that pharmaceutical policymaking perception is suboptimal among Lebanese early-career pharmacists^14^: pharmacists with higher self-efficacy and strategic thinking tend to have more positive perceptions of pharmaceutical policymaking and greater willingness to engage in policy-related roles. However, this study has primarily focused on direct associations rather than examining policymaking perceptions as a mediating construct that explains how individual competencies influence public service motivation.^14^ Furthermore, person-centered approaches, such as profile analysis, remain underutilized in pharmacy workforce research, particularly in Middle Eastern countries.^15^ These facts limit understanding of how combinations of competencies interact and influence motivational and professional outcomes, particularly among early-career pharmacists undergoing professional identity formation.

This background represents a critical conceptual gap, since theoretical frameworks such as social cognitive theory and motivational theories suggest that perceived competence and role relevance influence motivational orientations through cognitive appraisal mechanisms. Consequently, this study aimed to explore the relationship between personal and professional attributes, specifically leadership, strategic thinking, self-efficacy, and public service motivation among early-career pharmacists in Lebanon. The study further investigated the mediating effect of perceptions of pharmaceutical policymaking on the relationship between personal attributes and public service motivation and variations in these pathways across distinct pharmacist attribute profiles. Understanding these differences may help identify primary drivers and inform targeted strategies to foster public service engagement among early-career pharmacists.

## 2 Methods

### 2.1 Study design and sampling

This cross-sectional study was conducted from December 2023 to May 2024 using an electronic questionnaire, as part of a larger project exploring attitudes toward pharmaceutical policymaking and public service motivation among early-career pharmacists and pharmacy students in Lebanon. The study targeted early-career pharmacists, including first- to fourth-year students, who were completing their internships, and graduates with up to 10 years of professional practice.^16^ These participants were considered newly practicing pharmacists, as they share similar responsibilities, work environments, and professional stressors.

The online questionnaire was created using Google Forms and disseminated through the Lebanese Pharmacy Students' Association (LPSA), which shared the link with pharmacy students via WhatsApp, Facebook, and Instagram groups, as well as through available university email lists. Early-career pharmacists were reached through the same social media platforms.

To ensure data quality while using Google Forms, all essential questions were marked as mandatory, and response validation rules were applied to prevent entry errors. As a result, no training of data collectors was required, and there were no missing data or need for data imputation. All items were carefully worded to maximize clarity and reduce the risk of misinterpretation. Access to the form and the associated Google account was restricted to the principal investigator, and data were downloaded and stored securely offline. Given the questionnaire's length, it was deemed unlikely that the same participant would complete it twice. Participants received five reminder communications, and data collection proceeded until the targeted sample size was achieved.

Participation was voluntary and anonymous; therefore, the exact number of individuals approached or who declined participation could not be determined, and reasons for non-participation were not collected. At the beginning of the survey, a screening question verified whether respondents met the inclusion criterion of being early-career pharmacists (those who graduated within the past ten years). Early-career pharmacists represent a vital, rapidly evolving segment of the healthcare workforce, particularly in hospital, community, and clinical roles, and are estimated to number approximately 5000 pharmacists, according to the yearly number of graduates in Lebanon. Those who did not meet this criterion were automatically excluded and could not proceed with the questionnaire. Students were also reached through the Lebanese Pharmacy Students' Association (LPSA), while early-career pharmacists (those who graduated within the past ten years) were recruited via various social media platforms and available email lists from universities.

### 2.2 Ethics approval and consent to participate

The INSPECT-LB Research Ethics Committee approved the study protocol (approval number: 2023REC-013-INSPECT-10-09). At the beginning of the Google Form, participants were informed about the study's objectives and the voluntary nature of their participation, with the option to withdraw at any time. Anonymity and data confidentiality were maintained throughout data collection and analysis. Informed consent was obtained before participants could proceed, and those who did not agree were unable to access the survey.

### 2.3 Data collection tools

The questionnaire used in the current study was available in English and included sections on sociodemographic characteristics, education-related variables, and validated measures assessing leadership, self-efficacy, strategic thinking, policymaking, and public service motivation. The validated scales used to assess the key constructs were used with permission from the original authors. These instruments were originally developed and psychometrically validated in English. Because they have already undergone rigorous evaluation in terms of face validity, content validity, and construct validity in previous studies, no additional validation procedures were performed in the present study.

#### 2.3.1 The authentic leadership self-assessment questionnaire (ALSAQ-16)

This 16-item instrument evaluates authentic leadership using a 5-point Likert scale (from strongly agree to strongly disagree)^17^ across three dimensions: moral processing, self-awareness, and relational transparency. The scale has demonstrated high reliability (Cronbach alpha = 0.84). In the current study the Cronbach alpha value was excellent 0.933.

#### 2.3.2 The generalized self-efficacy scale (GSE-10)

The GSE is a 10-item self-reported measure designed to assess individuals' belief in their ability to cope with life's challenges and achieve successful outcomes, i.e., the belief that a person's actions are responsible for successful outcomes.^18^ Items are rated on a 4-point Likert scale, with higher scores indicating higher self-efficacy. The GSES has demonstrated strong internal consistency (commonly α = 0.80–0.90 across cultures) and robust construct validity, making it suitable for use in health professions research, education, and behavioral studies.^18^ In the current study the Cronbach alpha value was excellent 0.927.

#### 2.3.3 The research self-efficacy scale (RSES) - Short version

The RSES is commonly used to evaluate participants' confidence in their ability to conduct research tasks.^19^ This version comprises five items, with total scores ranging from 5 to 25, where higher scores reflect higher self-efficacy and confidence in research capabilities. The short version of the RSES was adapted from the original scale and has demonstrated good internal consistency, with Cronbach's alpha values commonly reported above 0.80 in prior studies.^19^, ^20^ In the current study, the Cronbach alpha value was excellent, 0.946.

#### 2.3.4 The strategic thinking questionnaire (STQ-15)

The STQ-15 tool assesses strategic thinking skills by asking the respondents to rate how often they apply these skills when faced with problems, dilemmas, or opportunities. Responses were recorded on a Likert scale, where 1 = rarely or almost never, 2 = once in a while, 3 = sometimes, 4 = often, and 5 = frequently or almost always, with higher scores representing greater use of cognitive skills.^21^ The STQ demonstrated acceptable psychometric properties in prior studies (internal consistency estimates typically above 0.80).^22^ In the current study, the Cronbach alpha value was excellent, 0.939.

#### 2.3.5 The public service motivation scale – short version (PSM-14)

The short version of Perry's PSM was used in this study^23^ across three dimensions, i.e., self-sacrifice, attraction to policymaking, and compassion. The scale has been widely used in various cultural and institutional settings, demonstrating its stability and relevance over time.^24^, ^25^, ^26^, ^27^ Responses were rated on a 5-point Likert scale from strongly disagree to strongly agree,^28^ with higher scores reflecting higher public service motivation. Studies using short PSM instruments find alpha values around or above 0.8, indicating acceptable internal consistency.^29^, ^30^ In the current study the Cronbach alpha value was excellent 0.959.

#### 2.3.6 The pharmaceutical policymaking perception scale (PPPS)

This scale, previously developed and validated as part of this project, was used to assess pharmaceutical policymaking attitudes.^14^ It comprises 12 items rated on a 5-point Likert scale from strongly disagree to strongly agree, with higher scores reflecting a better perception of pharmaceutical policymaking in Lebanon. The Cronbach's alpha for the PPPS scale, developed in a study among young pharmacists in Lebanon, was 0.926, indicating excellent internal consistency.^14^

### 2.4 Minimum sample size calculation

The sample size calculation was based on regression analysis with the Public Service Motivation (PSM) Scale as the dependent variable and the other scales as independent variables. The objective was not to predict one scale from the others but rather to examine associations with the PSM outcome. Therefore, the analytical approach and sample size calculation were specified accordingly.

The minimum required sample size was determined using G*Power version 3.0.10. For the omnibus test of multiple regression, a squared multiple correlation of R^2^ = 0.05 was assumed, corresponding to an effect size of 0.0526. With a 5% alpha error, 80% power, and 25 predictors in the model, the required sample size was *n* = 454. The target sample size was set at 500 participants to account for missing data and ensure sufficient power for three-variable path analysis mediation.^31^

### 2.5 Statistical analysis

The collected data were analyzed using SPSS (Statistical Package for Social Sciences) software, version 28.0. For descriptive analysis, frequency and percentage were used for categorical variables, and mean and standard deviation for quantitative variables. The distribution of these variables was considered normal using visual inspection of the histogram, while the skewness and kurtosis were lower than 2. These conditions are compatible with normality with a sample size higher than 100.^32^

For the bivariate analysis of continuous variables, the ANOVA was used to compare three groups or more after checking for homogeneity of variances using Levene's test. In case the variances are not homogenous, the Kruskal-Wallis test is used, respectively. After ANOVA and Kruskal-Wallis significant testing, post hoc analyses were conducted using Bonferroni adjustment. A Spearman correlation coefficient was used between continuous variables. In all cases, a *p*-value lower than 0.05 was considered significant.

Clusters were identified using k-means clustering based on participants' responses regarding their personal attributes (leadership, strategic thinking, global self-efficacy, and research self-efficacy). The clusters were constructed to be different (ANOVA significant), reflecting meaningful variations in participant profiles. Based on the cluster means, profiles were labeled as “strong,” “moderate,” or “low.” Specifically, Cluster 1 included participants with the highest mean personal attributes, Cluster 2 included those with moderate attributes, and Cluster 3 comprised participants with the lowest mean personal attributes. No formal validation of the clusters was performed, as this analysis was exploratory and intended to describe patterns within the data.

As for the multivariable analysis, multiple linear regressions were conducted to assess the correlates of each dependent variable in the whole sample, after checking the normality of the residuals, the linearity of the relationship, the absence of multicollinearity, and the homoscedasticity assumptions. The beta coefficient, its 95% Confidence Interval, and the *p*-value were reported. All the independent variables were introduced in the models, taking into account the maximum number allowed of variables to be included to assess the correlates of the dependent variable, based on the sample size^33^ (no less than 10 participants per independent variable): sociodemographic and other independent variables were added as appropriate.

The AMOS was used to calculate three pathways in the mediation analysis. While pathway A determined the regression coefficient for the effect of personal attributes on pharmaceutical policymaking attitude, pathway B examined the association between pharmaceutical policymaking attitude and public service motivation, and pathway C estimated the total and direct effect of personal attributes on public service motivation. The Macro generated bias-corrected bootstrapped 95% confidence intervals (CI) to test the significance of the indirect effect. Mediation was deemed significant when the CI around the indirect effect did not include zero. Two separate models were tested. In the first model, each personal attribute scale was entered independently as an explanatory variable. In the second model, the identified cluster groups were introduced as the independent variable. The use of integrated clustering and path analysis has already been described in the literature,^34^, ^35^ conditioned on the absence of circularity.

## 3 Results

Among 504 pharmacists, the majority were female and single, with 38.1% reporting no personal income. Also, 39.9% resided in Beirut (capital city), and 70.4% lived in urban areas. The distribution across universities was uneven, with the Lebanese International University (LIU), Beirut Arab University (BAU), and the Lebanese University (LU) being the most represented. Around 32% were early career pharmacists, 30% were senior pharmacy students (fifth and six year), and the remainder were in their fourth year or earlier pharmacy students. Additionally, 55.0% were students, 44.0% did not work, and 9.9% had completed postgraduate education. The mean age of participants was 23.81 ± 4.30 years. Further details are presented in Table 1.

**Table 1:** Descriptive characteristics of the studied sample (*N* = 504).

	Frequency (%)
**Gender**	
Male	146 (29.0%)
Female	358 (71.0%)
**Marital status**	
Single	434 (86.1%)
Married	66 (13.1%)
Divorced	3 (0.6%)
Widowed	1 (0.2%)
**Personal monthly income**	
No income	192 (38.1%)
<250 USD ($)	63 (12.5%)
250–500$	112 (22.2%)
500–1000$	99 (19.6%)
>1000$	38 (7.5%)
**Region of living**	
Beirut	201 (39.9%)
Mount Lebanon	91 (18.1%)
North	58 (11.5%)
South	86 (17.1%)
Beqaa	68 (13.5%)
**Living region category**	
Rural	149 (29.6%)
Urban	355 (70.4%)
**Current university**	
LU	96 (19.0%)
USJ	25 (5.0%)
BAU	103 (20.4%)
LAU	14 (2.8%)
LIU	245 (48.6%)
Foreign university	21 (4.2%)
**Current academic year**	
First	11 (2.2%)
Second	18 (3.6%)
Third	36 (7.1%)
Fourth	124 (24.6%)
Fifth	135 (26.8%)
Sixth	16 (3.2%)
Early career pharmacists	164 (32.6%)
**Highest level of education achieved**	
I am an undergraduate student	277 (55.0%)
Bachelor's degree	117 (23.2%)
PharmD	59 (11.7%)
Master's degree (MBA, MPH, etc.)	47 (9.3%)
Doctorate (PhD, DBA, etc.)	3 (0.6%)
**Employment status**	
Full-time employee	128 (25.4%)
Part-time employee	113 (22.4%)
Self-employed	41 (8.1%)
I do not work	222 (44.0%)
	**Mean ± SD**
**Age** (in years)	23.81 ± 4.30

**Abbreviations:** BAU: Beirut Arab University; DBA: Doctor of Business Administration; DPT: Doctor of Physical Therapy; GSES: General Self-Efficacy Scale; LAU: Lebanese American University; LIU: Lebanese International University; LU: Lebanese University; MBA: Master of Business Administration; MPH: Master of Public Health; PharmD: Doctor of Pharmacy; PhD: Doctor of Philosophy; USD: United States Dollar; USJ: Saint Joseph University.

Table 2 presents the classification of participants into three different clusters based on their personal attributes. Cluster 1 included participants with declared strong personal attributes. Cluster 2 included those with reported moderate personal attributes but low research self-efficacy. Cluster 3 comprised those with the lowest personal attributes but moderate research self-efficacy (Table 2).

**Table 2:** Classification of participants in the study sample by cluster analysis.

	Cluster 1 *N* = 211 (41.9%)	Cluster 2 *N* = 179 (35.5%)	Cluster 3 *N* = 114 (22.6%)	p-value*
Authentic Leadership Self-Assessment	63.27	56.25	47.74	<0.001
Generalized Self-Efficacy	23.21	18.76	17.42	<0.001
Research Self-Efficacy	86.05	54.41	76.02	<0.001
Strategic Thinking Questionnaire	59.32	48.93	41.62	<0.001

^⁎^Post hoc analysis significant for all (*p* < 0.05), except for Generalized Self-Efficacy [Cluster 2 and 3 (*p* > 0.05)].

Cluster 1 strong personal attributes. Cluster 2 moderate personal attributes but low research self-efficacy. Cluster 3 low personal attributes but moderate research self-efficacy.

The correlations between the personal and professional attributes of early-career pharmacists were displayed in Table 3. A positive and moderate association was found across all scores, ranging from 0.273 to 0.569 (*p* < 0.001). PSM showed positive correlations with leadership, generalized self-efficacy, research self-efficacy and strategic thinking (*r* = 0.331 to 0.569; *p* < 0.001). In contrast, Pharmaceutical Policymaking Perception showed very weak or non-significant correlations with most scales (ranging from *r* = −0.006, *p* = 0.885 to *r* = 0.155, p < 0.001).

**Table 3:** Spearman correlation coefficient between the scales used in the study.

	ALSAQ-16	GSE-10	RSES-11	STQ-15	PSM	PPPS
	Correlation coefficient	Correlation coefficient	Correlation coefficient	Correlation coefficient	Correlation coefficient	Correlation coefficient
**ALSAQ-16**	–					
**GSE-10**	0.465***	–				
*p-value*	**<0.001**					
**RSES-11**	0.273***	0.360***	–			
*p-value*	**<0.001**	**<0.001**				
**STQ-15**	0.489***	0.404***	0.322***	–		
*p-value*	**<0.001**	**<0.001**	**<0.001**	–		
**PSM**	0.569***	0.434***	0.331***	0.547***	**–**	
*p-value*	**<0.001**	**<0.001**	**<0.001**	**<0.001**	**–**	
**PPPS**	−0.006	0.109*	0.155***	0.117**	−0.039	–
*p-value*	0.885	**0.015**	**<0.001**	**0.009**	0.379	

* < 0.05; ** < 0.01; *** < 0.001. ALSAQ-16: Authentic Leadership Self-Assessment Questionnaire, GSE-10: The Generalized Self-Efficacy Scale, RSES-11: Research Self-Efficacy Scale short form, STQ-15: Strategic Thinking Questionnaire, PSM: Public Service Motivation, and PPPS: Pharmaceutical Policymaking Perception Scale.

Values marked in bold are significant (*p* < 0.05).

The multivariable analysis shows that none of the sociodemographic or educational variables were associated with public service motivation. Nevertheless, leadership, strategic thinking, and both generalized and research self-efficacy were positively associated with public service motivation while inversely correlated with pharmaceutical policymaking perception (Table 4).

**Table 4:** Multivariable analysis using the GLM method.

Dependent variable: Public Service Motivation score				
Independent variables	Reference group	Beta (95% CI) *	p-value	Significant (p < 0.05)
Gender (female vs male)	Male	−0.005 (−0.037; 0.026)	0.736	No
Highest education vs undergraduate	Undergraduate	0.018 (−0.030; 0.066)	0.456	No
BS education vs undergraduate	Undergraduate	0.020 (−0.022; 0.063)	0.346	No
Single vs others	others	−0.037 (−0.086; 0.012)	0.142	No
Income (high vs low)	low	−0.004 (−0.052; 0.044)	0.865	No
Income (middle vs low)	low	−0.0001 (−0.038; 0.038)	0.998	No
Age in years	–	−0.003 (−0.008; 0.001)	0.113	No
Authentic Leadership Self-Assessment	–	0.006 (0.004; 0.009)	**<0.001**	Yes
Generalized Self-Efficacy Scale	–	0.007 (0.003; 0.011)	**<0.001**	Yes
Research Self-Efficacy Scale short form	–	0.001 (0.0004; 0.002)	**0.004**	Yes
Strategic Thinking Questionnaire	–	0.007 (0.004; 0.009)	**<0.001**	Yes
Pharmaceutical Policymaking Perception Scale	–	−0.004 (−0.006; −0.002)	**<0.001**	Yes

Beta represents the regression coefficient for each independent variable.

95% CI = 95% confidence interval for the Beta value.

**Fig. 1:**
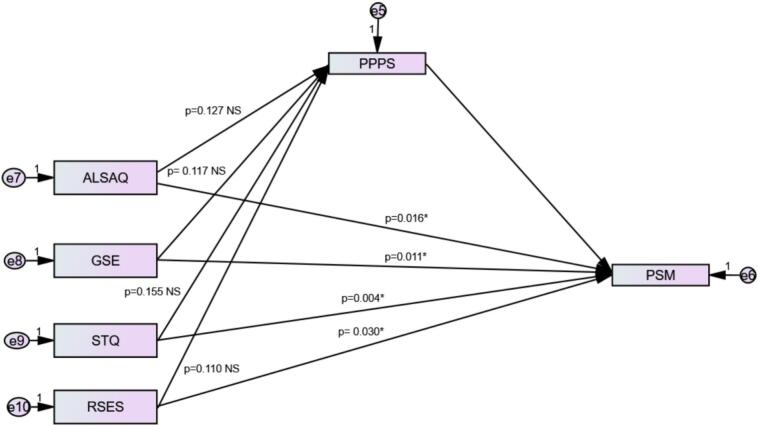
Mediation analysis of self-efficacy, leadership, and strategic thinking on Public Service Motivation mediated by Pharmaceutical Policymaking Perception. ALSAQ: Authentic Leadership Self-Assessment; GSE: Generalized Self-Efficacy; RSES: Research Self-Efficacy; STQ: Strategic Thinking Questionnaire, PSM: Public Service Motivation Scale; PPPS: Pharmaceutical Policymaking Perception Scale. *p-value less than 0.05 indicates a significant effect.

The mediation analysis was conducted using two models to examine the role of Pharmaceutical Policymaking Perception (PPPS) in the relationship between the of leadership and strategic thinking variables and Public Service Motivation (PSM). In Model 1, PPPS was tested as a mediator while considering each independent variable separately, with PSM as the dependent variable. The results indicated that the direct associations between each independent variable and PSM were statistically significant (*p* < 0.05 for all). However, the indirect effects through PPPS were not significant for any of the scales (all *p* > 0.05), suggesting no mediation effect in this model (Fig. 1).

In Model 2, the analysis was performed using the clustered group as the independent variable. The cluster group was significantly associated with PPPS (β = −2.216, *p* < 0.001), and PPPS was, in turn, positively associated with Public Service Motivation (β = 0.545, p < 0.001). Cluster belonging (reflecting incrementally weaker personal attributes) had a negative and statistically significant direct effect on Public Service Motivation (β = −5.071, *p* = 0.009). In addition, the indirect effect was also statistically significant (β = −1.209, *p* = 0.006), indicating that PPPS partially mediated the relationship between cluster group membership and Public Service Motivation (Table 5).

**Table 5:** Mediation analysis taking the Public Service Motivation Scale as the dependent variable and Pharmaceutical Policymaking as a mediating variable.

Predictor	Direct effect *p*-value	Indirect effect p-value	Mediation significance
Model 1: presents the mediation analysis conducted separately for each scale			
Authentic Leadership (ALSAQ-16)	**0.016**	0.127	NS
Generalized Self-Efficacy (GSE-10)	**0.011**	0.117	NS
Strategic Thinking (STQ-15)	**0.004**	0.155	NS
Research Self-Efficacy (RSES-11)	**0.030**	0.110	NS
Model 2: presents the mediation analysis conducted using the clustered group of scales			
Cluster group	**0.009**	0.006*	Partial mediation

NS = Not significant (p ≥ 0.05). *Partial significant mediation. Values marked in bold indicate a significant effect (*p* < 0.05).

## 4 Discussion

In the current study, personal and professional attributes of early-career pharmacists were generally positively associated, except for policymaking perception, which showed weak or no association with most traits. This finding raises questions about Lebanese pharmacists' satisfaction with current policymakers. Although no studies appear to have directly addressed this topic, some research on job satisfaction may indirectly support our findings. Public service motivation has been positively associated with overall job attitudes, job satisfaction, organizational commitment, and work engagement.^36^ Moreover, a global study among 1014 early-career pharmacists and pharmaceutical scientists from 92 countries found that job and career satisfaction ratings were negatively correlated with career expectations,^37^ which may partly explain our results.

In all analyses, leadership and public service motivation demonstrated a robust and independent relationship unaffected by potential mediators or confounding factors, including policymaking perception. This association has been supported in the literature,^1^, ^10^ which shows the importance of cultivating leadership and other personal attributes during early-career pharmacists' education. Such investment can enable them to assume leadership roles in practice settings at local, regional, and global levels. Leadership, strategic thinking, and self-efficacy were linked to higher public service motivation but lower perceptions of pharmaceutical policymaking. This finding suggests that early-career pharmacists with stronger personal attributes are more inclined to contribute to the profession in the future, despite expressing dissatisfaction with the current state of pharmaceutical policymaking. In the absence of similar studies in the literature, only indirect comparisons can help explain these results. For example, a study conducted in Lebanon reported a significant association between work satisfaction and management/leadership competencies.^38^ Additionally, a recent meta-analysis of 177 studies found that public service motivation was positively linked to overall job attitudes, job satisfaction, organizational commitment, and work engagement.^36^

Along the same line, early-career pharmacists with a high perception of pharmaceutical policymaking demonstrated a lower motivation toward public service, whereas those reporting stronger personal attributes had a lower perception of pharmaceutical policymaking. One possible explanation is lower satisfaction among more “knowledgeable” pharmacists with the general performance of pharmaceutical policymakers. This dissatisfaction is often linked to perceived undervaluation, inadequate compensation, and challenging work environments.^39^ Indeed, in recent decades, substantial problems have arisen in the pharmacy profession in Lebanon, where the practice of pharmacy and the preservation of pharmacists' professional standing have been the subject of several issues.^40^ Prior research has also revealed that pharmacists in Lebanon are dissatisfied with many aspects, such as pharmacy distribution, drug costs, profit margin, professional regulations, ethical prescription, the selling of fake medications, and political interference.^13^, ^41^, ^42^ Although these complex results require further investigation, there is a clear need to improve perceptions of policymaking while fostering public service motivation among early-career pharmacists.

Mediation analysis revealed no mediating effect of pharmaceutical policymaking perception between single personal attributes and public service motivation, possibly due to the small sample size or a genuine lack of correlation between the variables. However, when clustering personal attributes, cluster belonging was positively associated with public service motivation, and this relationship was partially mediated by perceptions of pharmaceutical policymaking. Cluster belonging, reflecting incrementally weaker personal attributes, was associated with significantly lower Public Service Motivation, as demonstrated by the negative and statistically significant direct effect. This indicates that individuals in clusters with weaker personal attributes tend to report lower motivation toward public service. The significant indirect effect through PPPS suggests that part of this association operates via PPPS, meaning that weaker personal attributes are linked to lower PPPS, which in turn is associated with reduced motivation. Because both the direct and indirect effects remained significant, PPPS partially mediates the relationship, indicating that additional factors beyond PPPS also contribute to the association between cluster belonging and Public Service Motivation. These results show the complex interplay between the studied concepts and the influential role of pharmaceutical policymakers among pharmacists who require, at least partially, a more favorable perception of pharmaceutical policymaking to become motivated for public service and work on optimizing the profession. A positive view of policymaking can foster motivation for public service, even when personal attributes are less developed.^43^ Given the limited literature on this topic, further research is needed to explore the relationship between research competence, policymaking perceptions, and public service motivation. This finding also shows the importance of addressing these aspects in parallel within educational institutions.

None of the sociodemographic or educational variables was associated with public service motivation. While the lack of association with sociodemographic characteristics warrants further investigation, the absence of a link with educational factors was noteworthy. This pattern suggests that formal education may have limited influence on pharmacists' personal and professional attributes, necessitating immediate action.^44^ In light of these findings, educational curricula should be revised to intentionally cultivate leadership, strategic thinking, and self-efficacy.

Complementary programs for continuing professional development and supportive work environments are also essential for consolidating pharmacists' competencies, aligning their personal and professional attributes, and empowering them to lead change within the profession. One additional notable concept that would deserve particular attention is altruistic awareness, which reflects the pharmacist's commitment to prioritizing patients' needs, creating a compassionate and respectful care environment, and encouraging patient autonomy. Altruistic awareness also involves beliefs, values, and attitudes that go beyond personal and organizational interests, focusing instead on the well-being of the broader community and promoting the public sense of responsibility and motivation for service.^1^, ^45^

### 4.1 Strengths and limitations

One of the study's strengths lies in the use of validated measures, which minimizes the risk of information bias and boosts the credibility of the results, while quantitative results favor “dose-effect” relationships. The application of multivariable analysis techniques further reduces the potential for confounding bias. Furthermore, the focus on recent data represents contemporary attitudes and patterns among Lebanon's early-career pharmacists.

However, some limitations should be acknowledged. The cross-sectional design precludes causality, while reliance on self-reported data might introduce information biases, including social desirability, recall, and confirmation biases. Also, the use of convenience sampling via social media may have reduced the generalizability of the findings. However, we attempted to increase sample heterogeneity by including participants with a broad range of personal and professional attributes. Additionally, the questionnaire's length and the online data collection process may have caused participant fatigue, potentially affecting response accuracy. The use of one comprehensive tool could help reduce participant burden. However, given the multifaceted nature of the phenomenon under study, we selected distinct, validated scales to capture the various dimensions with greater specificity and methodological rigor. This type of bias is expected to be non-differential, and non-significant correlations might be underestimated. Also, the exclusion of pharmacists who don't have internet access may limit the sample's representativeness, in addition to the non-participation of those who are not interested in the topic, which might induce a selection bias. Moreover, graduate, postgraduate, and practicing pharmacists could differ substantially in their backgrounds. As a result, examining their personal attributes in relation to public service motivation indicators could yield a heterogeneous conclusion. Future research should involve in-person interviews with a more diverse sample, including both early-career and more experienced pharmacists, to confirm these findings and address the unique challenges of the Lebanese context. Further exploration of educational, cultural, social, and economic variables is advised to optimize the profession and enhance pharmacists' engagement in policymaking and public service.

## 5 Conclusion

This research found that leadership, strategic thinking, and generalized and research self-efficacy were positively associated with public service motivation, while pharmaceutical policymaking was inversely related to it. Perception of pharmaceutical policymaking partially mediated the effect of weaker cluster membership on public service motivation. In view of the paucity of research in this area, conducting similar studies in diverse settings would help confirm the current results and further consolidate the causal relationships between these concepts. Meanwhile, education and research should prioritize early-career pharmacists' personal attributes to empower them in optimizing their profession and enhancing patient care.

## CRediT authorship contribution statement

**Chadia Haddad:** Writing – original draft, Formal analysis, Data curation. **Hala Sacre:** Writing – review & editing, Validation. **Jihan Safwan:** Writing – review & editing. **Deema Rahme:** Writing – review & editing. **Aline Hajj:** Writing – review & editing. **Jenny Elia:** Writing – review & editing, Project administration. **Joya El Ghawi:** Writing – review & editing, Project administration. **Lina Haidar:** Writing – review & editing, Project administration. **Lama Dimachkieh:** Writing – review & editing, Project administration. **Mahmoud Nasrallah:** Writing – review & editing, Project administration. **Sukaina Basma:** Writing – review & editing. **Pascale Salameh:** Writing – review & editing, Validation, Supervision, Conceptualization.

## Funding

No funding was received.

## Declaration of competing interest

The authors declare that they have no conflict of interest.

## References

[1] Belrhiti Z., Damme W.V., Belalia A., Marchal B. (2020). The effect of leadership on public service motivation: a multiple embedded case study in Morocco. BMJ Open.

[2] Nghi T.N., Thu H.T., Dinh T.T. (2022). The relationship between public service motivation, work enjoyment, and task performance: a preliminary study of healthcare workers in Vietnam. J Liberty Int Affairs.

[3] Schott C., Neumann O., Baertschi M., Ritz A. (2019). Public service motivation, prosocial motivation and altruism: towards disentanglement and conceptual clarity. Int J Public Adm.

[4] Droege M., Assa-Eley M.T. (2005).

[5] Camilleri E. (2007). Antecedents affecting public service motivation. Pers Rev.

[6] Crafford L., Wouters A., Bronkhorst E., Gous A.G.S., Kusurkar R.A. (2021). Exploring factors associated with the motivation of clinical pharmacists: a focus on the south African context. Front Med.

[7] Luetsch K. (2017). Attitudes and attributes of pharmacists in relation to practice change – a scoping review and discussion. Res Social Adm Pharm.

[8] Fernandes A., Santinha G., Forte T. (2022). Public service motivation and determining factors to attract and retain health professionals in the public sector: a systematic review. Behav Sci.

[9] Jung G., Moon K.-K. (2024). Examining public service motivation’s impact on organizational commitment: focusing on moderating roles of hygiene and motivation factors. Behav Sci.

[10] Hameduddin T., Engbers T. (2022). Leadership and public service motivation: a systematic synthesis. Int Public Manag J.

[11] BinDhim N.F., Althumiri N.A., Albluwi R.A., Aljadhey H.S. (2024). Competencies, skills, and personal characteristics needed for pharmacy leaders: an in-depth interview. Saudi Pharm J.

[12] Sacre H., Hamra R., Hassoun C. (2023). Towards a national pharmaceutical strategy in Lebanon: ensuring access to quality and safe medications for all. Pharm Educ.

[13] Hajj A., Zeenny R.M., Sacre H., Akel M., Haddad C., Salameh P. (2023). Pharmacy education and workforce: strategic recommendations based on expert consensus in Lebanon. J Pharm Policy Pract.

[14] Haddad C., Sacre H., Safwan J. (2025).

[15] Aman M., Arakawa N., Anderson C. (2025). Leadership competencies and behaviours in pharmacy: a qualitative content analysis. Res Social Adm Pharm.

[16] Pharmaceutical Society of Australia Early Career Pharmacists (2018). Pharmaceutical Society of Australia. https://www.psa.org.au/early-career-pharmacists/.

[17] Panczyk M., Jaworski M., Iwanow L., Cieślak I., Gotlib J. (2019). Psychometric properties of authentic leadership self-assessment questionnaire in a population-based sample of polish nurses. J Adv Nurs.

[18] Schwarzer R., Jerusalem M., Weinman J., Wright S.C., Johnston M. (1995). Measures in Health Psychology: A User’s Portfolio. Causal and Control Beliefs.

[19] Yasmin F., Schultz A., Phiri A., Weigel R. (2023). “I need to be the first one with a different approach and to make a difference to the people”: a mixed methods pilot study on non-physician clinicians training in Malawi. AMEP.

[20] Bates I., Ansong D., Bedu-Addo G. (2007). Evaluation of a learner-designed course for teaching health research skills in Ghana. BMC Med Educ.

[21] Pisapia J., Morris J., Cavanaugh G., Ellington L. (2011).

[22] Rodrigues R.I., Neves J., Ferreira A. (2021). Strategic thinking: the construction and validation of an instrument. Pol Psychol Bull.

[23] Perry J.L. (1996). Measuring public service motivation: an assessment of construct reliability and validity. J Public Adm Res Theory.

[24] Budiyanti H., Patiro S.P.S., Yamin A. (2019). Public service motivation measurement: a test of Perry’s scale in Indonesia. JKAP (Jurnal Kebijakan Dan Administrasi Publik).

[25] Coursey D.H., Pandey S.K. (2007). Public service motivation measurement: testing an abridged version of Perry’s proposed scale. Administr Soc.

[26] Meyer-Sahling J.-H., Mikkelsen K.S., Schuster C. (2019). The causal effect of public service motivation on ethical behavior in the public sector: evidence from a large-scale survey experiment. J Public Adm Res Theory.

[27] Zhang H., Zhang Q., Huang G. (2022). Analysis of evaluation dimensions of public service motivation of Chinese college students—qualitative study based on grounded theory. Int J Environ Res Public Health.

[28] Gan K., Li L., Wang Q. (2013).

[29] Coursey D.H., Perry J.L., Brudney J.L., Littlepage L. (2008). Psychometric verification of Perry’s public service motivation instrument: results for volunteer exemplars. Rev Publ Person Administr.

[30] Kim S. (2009). Revising Perry’s measurement scale of public service motivation. Am Rev Public Adm.

[31] Sim M., Kim S.-Y., Suh Y. (2022). Sample size requirements for simple and complex mediation models. Educ Psychol Meas.

[32] Ghasemi A., Zahediasl S. (2012). Normality tests for statistical analysis: a guide for non-statisticians. Int J Endocrinol Metab.

[33] Harrell F.E. (2015).

[34] Muthén B., Muthén L.K. (2000). Integrating person-centered and variable-centered analyses: growth mixture modeling with latent trajectory classes. Alcohol Clin Exp Res.

[35] Howard M.C., Hoffman M.E. (2018). Variable-centered, person-centered, and person-specific approaches: where theory meets the method. Organ Res Methods.

[36] Tang H., An S., Zhang L., Xiao Y., Li X. (2024). The antecedents and outcomes of public service motivation: a meta-analysis using the job demands–resources model. Behav Sci.

[37] Meilianti S., Matuluko A., Ibrahim N., Uzman N., Bates I. (2022). A global study on job and career satisfaction of early-career pharmacists and pharmaceutical scientists. Explor Res Clin Soc Pharm.

[38] Zeineddine L., Sacre H., Haddad C. (2023). The association of management and leadership competencies with work satisfaction among pharmacists in Lebanon. J Pharm Policy Pract.

[39] Cherecheș M.C., Finta H., Prisada R.M., Rusu A. (2024). Pharmacists’ professional satisfaction and challenges: a netnographic analysis of Reddit and Facebook discussions. Pharmacy.

[40] Alameddine M., Bou Karroum K., Hijazi M.A. (2019). Upscaling the pharmacy profession in Lebanon: workforce distribution and key improvement opportunities. Hum Resour Health.

[41] Iskandar K., Hallit S., Raad E.B., Droubi F., Layoun N., Salameh P. (2017). Community pharmacy in Lebanon: a societal perspective. Pharm Pract (Granada).

[42] Salameh P., Hamdan I. (2007). Pharmacy manpower in Lebanon: an exploratory look at work-related satisfaction. Res Social Adm Pharm.

[43] Darmayanti, Perizade B, Isnurhadi, Yuliani (2024). Public service motivation as an intervening variable of self-efficacy and competency towards state civil apparatus performance. KSS.

[44] Wang X., Wen L., Fu H., Yin Z. (2024). Exploring the motivation of self-directed learning of hospital pharmacists: a multicentre qualitative study. BMJ Open.

[45] Allinson M., Chaar B. (2019). How to demonstrate empathy and compassion in a pharmacy setting. Pharm J.

